# Soft Tissue Sarcoma in Sub‐Saharan Africa: Surgical Management Outcomes, Gaps, and Future Prospects: A Narrative Review

**DOI:** 10.1002/hsr2.70215

**Published:** 2024-11-25

**Authors:** Favour Tope Adebusoye, Sakshi Roy, Hareesha Rishab Bharadwaj, Syed Hasham Ali, Wireko Andrew Awuah, Tomas Ferreira, Joecelyn Kirani Tan, Nicholas Aderinto, Saleha Azeem, Paul Olutosin Salako, Pearl Ohenewaa Tenkorang, Harendra Kumar, Toufik Abdul‐Rahman

**Affiliations:** ^1^ Faculty of Medicine Sumy State University Sumy Ukraine; ^2^ School of Medicine Queen's University Belfast Belfast UK; ^3^ Faculty of Biology Medicine and Health The University of Manchester Manchester UK; ^4^ Faculty of Medicine Dow University of Health Sciences Karachi Pakistan; ^5^ School of Clinical Medicine University of Cambridge Cambridge UK; ^6^ Faculty of Medicine University of St Andrews St. Andrews Scotland UK; ^7^ Department of Medicine and Surgery Ladoke Akintola University of Technology Ogbomoso Nigeria; ^8^ Faculty of Medicine King Edward Medical University Lahore Pakistan; ^9^ Faculty of Medicine V.N Karazin National University Kharkov Kharkiv Ukraine; ^10^ Faculty of Medicine University of Ghana Medical School Accra Ghana

**Keywords:** oncology, soft tissue sarcoma, sub‐Saharan Africa, surgery

## Abstract

**Background and Aims:**

The diagnosis and management of soft tissue sarcoma (STS) in sub‐Saharan Africa (SSA) present significant challenges. Positive outcomes in STS treatment include achieving negative margins, improved quality of life, and reduced recurrence rates, while negative outcomes involve incomplete resection, local recurrence, and surgical complications. This review aims to examine the current state of STS management in SSA, identify key challenges, and propose potential solutions to improve patient outcomes.

**Methods:**

A comprehensive review of the literature using PubMed/MEDLINE, Google Scholar, and Scopus, focusing on English‐language studies examining the management of STS in SSA. Inclusion criteria centered on studies reporting on surgical interventions, outcomes, and healthcare challenges in the region. Articles lacking sufficient data, non‐English sources, conference abstracts, and duplicates were excluded.

**Results:**

Findings highlight several obstacles in the management of STS in SSA, including limited multidisciplinary teams, inadequate healthcare infrastructure, financial constraints, and lack of standardized treatment protocols. Key themes such as diagnostic capacity and resource allocation were identified as significant barriers.

**Conclusion:**

Improving STS outcomes in SSA requires investment in healthcare infrastructure, professional development, enhanced funding, and collaborative research. Addressing these gaps is crucial to achieving better surgical management and improving survival rates for patients with STS in SSA.

## 1 Introduction

Soft tissue sarcoma (STS) is a complex malignancy originating from various soft tissues, including muscles, tendons, fat, and blood vessels. Notable examples include leiomyosarcoma, liposarcoma, and synovial sarcoma [1, 2, 3, 4]. The presence of these heterogeneous tissue types contributes to the intricate biological behavior and anatomical complexity observed in STS cases [1]. STS exhibits variability in behavior, with differences in growth patterns, invasiveness, and potential for metastasis [1, 2, 3]. Accurate diagnosis involves imaging techniques and biopsies, while treatment options encompass surgery, radiation therapy (RT), chemotherapy, targeted therapies, and immunotherapy [1]. The choice of treatment depends on factors such as tumor size, location, stage, and the patient's overall health [1, 2, 3, 4, 5].

STS is a rare tumor, occurring in approximately 1–2 out of every 100,000 individuals worldwide [1]. However, the incidence of STS in sub‐Saharan Africa (SSA) exhibits gender‐based variability. Studies conducted in Nigeria estimated rates of approximately 0.8 cases per 100,000 individuals for males and 0.5 cases per 100,000 individuals for females [6]. Nonetheless, the lack of comprehensive cancer registries and diagnostic facilities in these countries hinders the accurate assessment of the true prevalence and incidence rates of STS in the region [6]. Despite these limitations, STS poses a significant health concern in SSA, leading to complications, reduced quality of life, high mortality rates, as well as diagnostic and management challenges [7, 8, 9, 10].

Management of STS typically involves surgical resection with perioperative RT as the standard of care [11]. However, the limited availability of radiotherapy services in SSA and the uncertain role of chemotherapy for managing various subtypes of STS have positioned surgery as the primary treatment modality, with accurate diagnosis by pathologists being crucial for effective surgical management [2, 3, 11, 12, 13]. The absence or scarcity of radiotherapy facilities poses a significant challenge to delivering comprehensive cancer care [13]. Consequently, surgery assumes an increasingly important role, offering the best opportunity to achieve local control by ensuring R0 resection [2, 5, 8, 9]. Moreover, factors such as cost‐effectiveness, the presence of basic surgical equipment in hospitals, and the need for prompt intervention in specific cases further underscore the importance of surgery in these settings [7, 9]. Given the concerns about toxicity to adjacent structures and the effectiveness of chemotherapy, surgical resection emerges as the main focus for managing STS in SSA [14, 15].

With this context in mind, the aim of this paper is to elucidate the surgical outcomes of STS, identify existing management gaps, and provide recommendations for improving the diagnosis, treatment, and overall care of STS in SSA.

## 2 Methods

This article offers a comprehensive examination of the management of STS in SSA. We conducted an extensive review of literature and empirical evidence to explore outcomes, challenges, and future prospects for STS treatment in the region.

We reviewed various studies, reports, and articles using databases such as PubMed/MEDLINE, Google Scholar, and Scopus, applying qualitative analysis to identify key themes and insights. Our inclusion criteria focused on English‐language sources addressing STS management and outcomes in SSA, while excluding studies with insufficient data, conference abstracts, opinion pieces, duplicate records, and non‐English sources.

Our search included terms like “soft tissue sarcoma,” “surgical management,” “SSA,” and “treatment outcomes,” and examined indicators such as “surgical interventions” and “financial constraints.” We also reviewed references from recent reviews to ensure a comprehensive assessment. Methodological details are summarized in Table 1.

**Table 1:** Summary of methodology.

Methodology steps	Description
Literature search	‐ PubMed/MEDLINE, Google Scholar, Scopus
Inclusion criteria	‐ Full‐text articles published in English. ‐ Studies addressing the management and outcomes of STS in SSA.
Exclusion criteria	‐ Conference abstracts, opinion pieces, and duplicate records. ‐ Studies lacking sufficient data or relevance to the topic. ‐ Non‐English language studies.
Search terms	‐ Keywords such as “soft tissue sarcoma,” “surgical management,” “Sub‐Saharan Africa,” and “treatment outcomes” were used in combination with indicators like “surgical interventions” and “financial constraints”
Additional search criteria	‐ Supplementary sources were identified by manually examining references cited in recent reviews focused on the topic

Abbreviations: SSA = sub‐Saharan Africa, STS = soft tissue sarcoma.

## 3 Surgical Management Outcomes of STS in SSA

### 3.1 Positive Outcomes

#### 3.1.1 Resection

Pathologically negative margins, known as R0 resection, play a crucial role in STS surgery as they indicate the complete removal of the tumor, thereby reducing the risk of local recurrence. The achievement of R0 resection has been consistently observed in various studies. For instance, Ayandipo and colleagues reported that 88% of patients achieved R0 margins, aligning with similar findings from studies conducted in Europe and North America [6, 16, 17, 18]. Additionally, other investigations have also reported complete excision in their respective studies [2, 3, 5, 7, 10, 12, 14, 19]. These consistent findings emphasize the significance of optimal resection margins in the surgical management of STS.

#### 3.1.2 Improvement After Surgery

The postsurgical period plays a vital role in facilitating the restoration of normal functions and improving overall well‐being for patients. This is evident in SSA, where surgical interventions have been shown to enhance quality of life and provide symptom relief, aligning with findings from global studies [18, 20]. Chauke and colleagues reported significant improvements in the quality of life of a patient with ameloblastic fibrosarcoma following the surgical excision of a jaw mass and subsequent reconstructive procedures [12]. Additionally, other studies have also observed patient satisfaction with functional outcomes [5, 21]. These findings underscore the significance and effectiveness of surgical interventions in improving outcomes for patients with STS.

#### 3.1.3 Survival and Recurrence

Survival and recurrence are key determinants of the prognosis and long‐term outcomes for patients with STS. Studies investigating surgical management have consistently reported favorable outcomes concerning survival and recurrence rates. Significant extensions in survival periods have been observed among patients who underwent surgery [9, 19]. Notably, patients undergoing surgery experienced high survival rates and relatively short healing times [22]. This positive trend was further supported by Uba and colleagues, who identified a substantial reduction in recurrence rates [7]. These findings underscore the positive impact of surgical interventions on survival outcomes and the potential to reduce recurrence rates in STS patients within the region. The positive outcomes are summarized in Table 2.

**Table 2:** Surgical outcomes of soft tissue sarcoma management in sub‐Saharan African Countries.

References	Histological tumor type(s) in studies	Location	Surgical intervention	Positive findings	Negative findings/complications
Uba and Chirdan [7] (Nigeria)	Alveolar rhabdomyosarcoma, Embryonal rhabdomyosarcoma	Lower limbs, upper limbs, head, neck, orbit, perineal, perianal	Primary excision, second‐look operation, biopsy	‐ ⁠Two patients underwent successful primary surgery with wide resection, resulting in complete excision of the tumor ‐ ⁠Seven out of 11 patients who underwent primary excision, the tumor was completely removed ‐ ⁠One patient with a local recurrence underwent re‐excision, which effectively addressed the issue	‐ A total of 13 (72.2%) patients died during treatment, including 10 who had operative treatment and 3 who declined surgical treatment ‐ ⁠The mortality rate was 83.3% (5/6) for patients with embryonal rhabdomyosarcoma and 66.7% (8/12) for those with alveolar rhabdomyosarcoma ‐ Four out of 11 patients who underwent primary excision had some residual tumors remaining after surgery ‐ Three patients who had surgical treatment still had inguinal node involvement and required ongoing chemotherapy
Afuwape et al. [9] (Nigeria)	GISTs	Stomach	Total gastrectomy, transverse colectomy, splenectomy, distal pancreatectomy, loop esophagojejunal anastomosis, Jejunojejunostomy	‐ Patient was symptom‐free 3 months after surgery	‐ ⁠Patient died 8 months after surgery
Fasunla and Daniel [10] (Nigeria)	Embryonal rhabdomyosarcoma, Alveolar rhabdomyosarcoma, Botryoid rhabdomyosarcoma, Myxofibrosarcoma, Kaposi's sarcoma, Peripheral nerve sheath tumor, Synovial cell sarcoma, Malignant fibrous histiocytoma	Ear, cervico‐facial, sinonasal, larynx, cervico‐facial, neck, face parapharyngeal, oropharynx	Tumor resection, neck dissection, orbital exenteration, total maxillectomy, craniotomy	‐ One patient with sinonasal alveolar rhabdomyosarcoma had localized disease that was completely resected with clear margins	‐ 77.8% of patients died 1 year after diagnosis ‐ ⁠Recurrence was observed in l patient
Motanya and Saidi [3] (Kenya)	Fibrosarcoma, Rhabdomyosarcoma, Neurofibrosarcoma, Alveolar soft tissue sarcoma, Myxofibrosarcoma, Hemangiosarcoma, Hemangiopericytoma, Pleomorphic sarcoma, PNET, Malignant peripheral nerve sheath tumor	Lower limb, upper limbs, retroperitoneal/intraperitoneal, trunk, head/neck	Wide or radical excision, amputation or disarticulation excision	‐ Negative histological surgical margins were observed in 44.0% of patients	‐ Patients with positive histological surgical margins were 56.0% ‐ 78.0% of patients developed recurrences, (51.3% of cases were local)
Chauke et al. [12] (South Africa)	Ameloblastic fibrosarcoma	Oral cavity	Wide local excision, lower lip salvage segmental mandibulectomy, reconstructive plate fitted based on 3D model of resection, free anterolateral thigh flap for intraoral lining	‐ Complete tumor resection as judged grossly ‐ Ability to feed orally restored, with good aesthetic progress at 6 months	‐ Postoperative complication, including, lip commissure dehiscence, malocclusion, oral incontinence, reconstructive plate exposure and delayed decannulation of the tracheostomy ‐ Intraoral flap dehiscence recurred three times
Sidy et al. [2] (Nigeria)	Dermatofibrosarcoma protuberans, alveolar rhabdomyosarcoma, angiosarcoma, leiomyosarcoma, fibrosarcoma, neurofibrosarcoma, epithelioid sarcoma, pleomorphic cell sarcoma	Leg, thigh, groin, axilla	Conservative surgery, lymph node dissection, thigh amputation, upper limb disarticulation, reconstructive surgery (rotation flaps, skin graft)	‐ R0 resection was observed in 50% of patients	‐ ⁠Five patients were dead after 10 months follow‐up ‐ Four patients had recurrences
Ayandipo et al. [6] (Nigeria)	Liposarcoma, leiomyosarcoma, malignant fibrous histiocytoma, fibrosarcoma, synovial sarcoma, malignant peripheral nerve sheath tumor, angiosarcoma, phyllodes, embryonal rhabdomyosarcoma, epithelioid sarcoma, GISTs	Upper extremity, lower extremity, retroperitoneal, truncal, head and neck, visceral	Surgical resection (with neo or adjuvant chemo radiotherapy)	‐ Grossly negative tumor resection margin was observed in 88% of patients	‐ Postoperative complications occurred in 18.7% of patients, including seroma, flap dehiscence, necrosis, and wound ‐ ⁠Local recurrence was observed in 17% of patients ‐ ⁠Positive resection margins in 12% of patients, particularly in retroperitoneal and truncal STS ‐ ⁠Postoperative complications occurred in 18.2% of patients, including surgical site infections, skin grafting failure, wound gaping/dehiscence, stump necrosis, phantom pain
Ayandipo et al. [21] (Nigeria)	Fibrosarcoma, dermatofibrosarcoma protuberans	Anterolateral aspect of left leg (above lateral malleolus), Anterolateral aspect of right leg (above lateral malleolus)	Wide local excision (with soft tissue reconstruction), wide local excision (with soft tissue cover)	‐ Patients were satisfied with the functional outcome	Not reported
Brown et al. [14] (Nigeria)	Rhabdomyosarcoma	Orbit, check, neck, vagina, paratesticular region, nasopharynx, bladder, pelvis, extremities	Tumor excision, debulking surgery	‐ Complete excision was observed in seven patients	‐ Not reported
Gbessi et al. [5] (Republic of Benin)	GISTs	Stomach, large bowel, small intestine, omentum, mesentery	Tumor resection performed during open surgery	‐ Complete (R0) tumor resection	‐ Recurrence observed in two patients
Sithole et al. [19] (South Africa)	GISTs	Stomach, extra‐gastric (rectum)	Multivisceral resection, metastatic resection, liver resection, abdominoperineal resection, gastric resection (with imatinib)	‐ R0 resection margins for the primary disease and metastases ‐ ⁠Patients treated by surgery (with imatinib) had survival period of greater than 11 years	‐ Three patients died after surgery. ‐ One patient died 8 days after surgery ‐ ⁠Two patients died within the fourth and fifth months of surgery from respiratory complications ‐ Recurrence observed in one patient after 1 year
Zongo et al. [22] (Burkina Faso and Guinea)	Dermatofibrosarcoma	Thorax, abdomen, limbs, head	Tumor excision, wide resection, resection with flap coverage, skin grafting, skin coverage by flaps	‐ 97.3% survival rate after surgery ‐ Healing time was 14 + 7 days for healing by primary intention with extremes of 12 and 33 days	‐ Reoperation was reperformed in all cases ‐ ⁠Recurrences in 16.4% of patients ‐ Healing delayed by (22 ± 5) days for patients with skin flap

Abbreviations: GIST = gastrointestinal stromal tumor, PNET = primitive neuro‐ectodermal tumor.

### 3.2 Negative Outcomes

#### 3.2.1 Complete Resection Challenges and Local Recurrence

Achieving complete resection, defined as the removal of the entire tumor with R0 margins, remains a significant challenge in the surgical management of STS. Ayandipo and colleagues reported positive resection margins in 12% of patients, predominantly observed in cases of retroperitoneal and truncal STS [6]. Similarly, Uba and colleagues identified residual tumors in a substantial proportion (4 out of 11) of patients who underwent primary excision, indicating disease persistence despite surgical intervention [7].

Furthermore, local recurrence, defined as the reappearance of the tumor at or near the original tumor site, poses a significant issue following surgical management. Ayandipo and colleagues reported a local recurrence rate of 17% among STS patients, even in cases where R0 resection was achieved [6]. This suggests that despite efforts to achieve complete resection, recurrence remains a challenge, possibly due to the lack of radiotherapy [4]. Motanya and colleagues observed recurrence in 78.0% of patients, with 51.3% representing local recurrences [3]. Additionally, studies have noted the presence of residual tumor following primary excision, highlighting the ongoing disease burden despite surgical intervention [7].

#### 3.2.2 Postoperative Complications and Surgical Mortality

Surgical procedures for STS in SSA are not exempt from postoperative complications. Ayandipo and colleagues documented complications in 18.7% of patients, including seroma, flap dehiscence, necrosis, and wound‐related issues [6]. Similarly, Chauke and colleagues reported complications such as lip commissure dehiscence, malocclusion, oral incontinence, reconstructive plate exposure, and delayed decannulation of the tracheostomy [12]. These complications underscore the importance of meticulous perioperative management to minimize adverse outcomes.

In addition, mortality is also associated with the surgical management of STS. Sithole and colleagues documented the death of three patients, one shortly after the procedure and two within the fourth and fifth months due to respiratory complications [19]. Moreover, studies have reported alarming mortality rates of 66.7% and 72.2% among patients with alveolar and embryonal rhabdomyosarcomas, respectively [7]. These findings emphasize the potential risks and complications associated with surgical interventions for STS in SSA.

While surgical management is indispensable in treating STS, the evidence reveals significant challenges and adverse outcomes in SSA. Factors such as positive resection margins, residual tumors, high local recurrence rates, postoperative complications, and surgical mortality highlight the urgent need for innovative approaches and comprehensive management strategies. It is imperative to address these limitations and strive for improved outcomes in the surgical management of STS in the region. The negative outcomes are summarized in Table 2.

### 3.3 Surgical Management Gaps of STS in SSA

The effective management of STS requires the active involvement of comprehensive multidisciplinary teams (MDTs) and supportive healthcare professionals. However, an examination of existing literature on the surgical management of STS in SSA reveals notable management gaps that need to be addressed. This section aims to explore and discuss these gaps within the context of SSA, shedding light on the challenges and limitations faced in effectively managing STS in the region.

#### 3.3.1 Limited Availability of Multidisciplinary Teams and Insufficient Workforce

MDTs play a crucial role in the surgical management of STS by providing a collaborative and multidimensional approach that optimizes diagnosis, treatment planning, and overall patient care. However, the availability of such teams is limited in SSA, leading to inadequacies in STS management [12]. The absence of a well‐functioning MDT comprising surgical oncologists, reconstructive surgeons, pathologists, and other healthcare professionals may result in suboptimal surgical outcomes [21].

Inadequate workforce further exacerbates the challenges in STS surgical management. As the incidence of STS rises, there is an urgent need for an adequate number of surgical oncologists to provide timely and effective interventions [21]. However, an insufficient workforce contributes to delays in diagnosis, limited treatment options, and compromised patient outcomes.

#### 3.3.2 Financial Constraints, Limited Healthcare Facilities, and Inadequate Infrastructure

Socioeconomic factors significantly impact the surgical management of STS in SSA. Financial limitations pose a significant barrier, as many individuals lack the necessary resources to afford medical expenses, including consultations, diagnostic tests, surgical procedures, and postoperative care. Studies have highlighted the impact of socioeconomic factors on STS management in SSA, with high treatment abandonment rates due to financial constraints [14]. Limited financial resources restrict access to STS surgical management, impeding optimal decision‐making, and compromising treatment outcomes [2].

Limited access to healthcare facilities and inadequate infrastructure further compound the problem. Patients face challenges in accessing specialized centers for surgical management, including advanced imaging techniques, targeted therapies, and specialized surgical techniques. Resource limitations in SSA contribute to disparities in access to quality surgical care, particularly in remote or rural regions where geographical barriers and a lack of healthcare facilities hinder timely interventions [2, 12, 14].

#### 3.3.3 Limited Diagnostic Capacity

Diagnostic capacity is a significant challenge in STS surgical management. A paucity of immunohistochemical studies in resource‐challenged environments results in misdiagnoses and underreporting of STS cases. Limited diagnostic capabilities hinder accurate and timely diagnosis, affecting the implementation of appropriate treatment strategies [10]. The lack of early diagnosis and referral due to limited skilled personnel and delayed processing of biopsy specimens impedes effective management [12]. Furthermore, insufficient resources for advanced imaging techniques and timely histopathological diagnosis exacerbate the diagnostic gaps. These limitations in diagnostic capabilities pose a significant challenge to accurate and timely diagnosis, hindering appropriate treatment strategies.

#### 3.3.4 Lack of Treatment Guidelines and Insufficient Postoperative Rehabilitation and Supportive Care

Research and innovation gaps contribute to the challenges in STS surgical management. The absence of clear treatment guidelines for specific STS cases, such as ameloblastic fibrosarcoma and gastrointestinal stromal tumor (GIST), leads to variations in management and potentially suboptimal outcomes [5, 12]. The absence of clear guidelines for reconstructive surgery timing and limited access to specialized surgical techniques raise concerns regarding optimal surgical interventions. Additionally, the lack of consensus on the role of adjuvant therapy and the absence of long‐term surveillance protocols further contribute to the management gaps in SSA [5, 10, 14, 19, 22].

**Figure 1:**
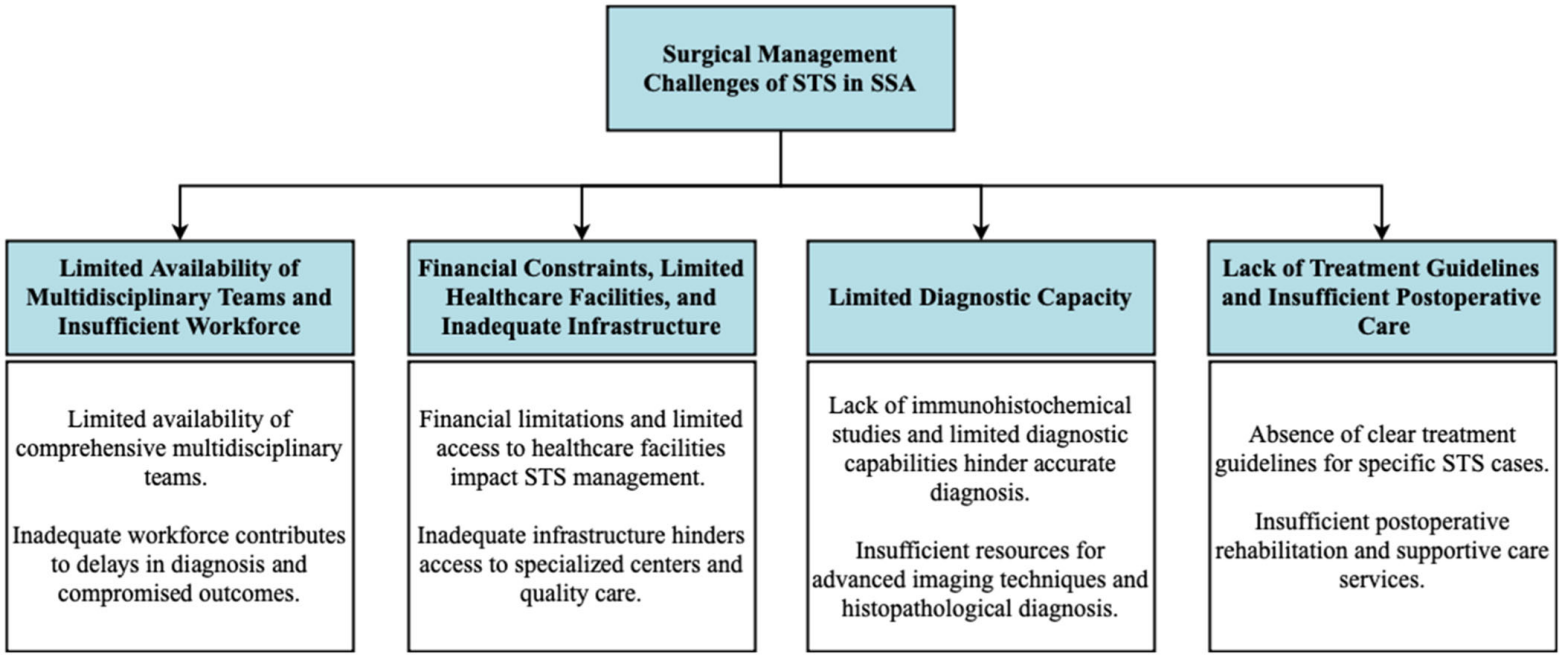
Surgical management challenges of STS in SSA.

Moreover, insufficient postoperative rehabilitation and supportive care significantly impact treatment outcomes. The absence of comprehensive care services such as physical therapy, pain management, psychosocial support, and survivorship care can lead to diminished quality of life and hinder long‐term recovery (Fasunla and colleagues; Brown and colleagues). These gaps in postoperative care may result in increased complications, prolonged recovery times, and potentially worse overall treatment outcomes for patients with STS [10, 14]. As a consequence, patients may experience reduced functional abilities and a lower quality of life, which emphasizes the need for improved supportive care strategies to enhance long‐term recovery and survival. Surgical management gaps are summarized in Figure 1.

## 4 Recommendations and Future Prospects

To enhance the surgical management of STS in SSA, several targeted recommendations can be implemented. First, establishing continuous professional development programs is crucial. These should offer specialized training and workshops tailored to local challenges, similar to initiatives by AO Alliance and Global Surgery Foundation (GSF), which enhance surgical care in resource‐limited settings [23]. In addition, strengthening funding networks and fostering international collaborations is essential. For example, Stanford University collaborates with the Ministries of Health in Zambia and Rwanda to increase surgical capacity and infrastructure through National Surgical Health Plans [24]. Such efforts are vital for improving STS management in SSA.

Investing in healthcare infrastructure is a critical step towards improving surgical management. Adequate resources should be allocated to developing and upgrading surgical facilities, acquiring essential imaging equipment, and ensuring access to necessary laboratory resources. By enhancing infrastructure, the capacity to deliver high‐quality surgical care can be significantly improved. Additionally, to address limited diagnostic capacity in SSA, targeted strategies are essential. Enhancing laboratory infrastructure through initiatives like those by the African Society for Laboratory Medicine can provide advanced diagnostic tools and equipment [25]. Investing in training programs for healthcare professionals, such as those exemplified by the Uganda Cancer Institute's (UCI) efforts, helps build local expertise [26]. Strengthening telemedicine and remote diagnostic support can also bridge gaps in areas with limited access to specialists. These combined efforts are crucial for advancing diagnostic capabilities and improving patient outcomes.

Regular and systematic surgical audits should be conducted to identify areas for improvement and ensure quality assurance in surgical management. These audits would provide valuable insights into performance monitoring, identification of best practices, and the overall enhancement of surgical care. Adopting a multidisciplinary approach is crucial for optimizing STS surgical management. Effective collaboration among surgical oncologists, reconstructive surgeons, pathologists, and other specialists is essential for improving preoperative planning, surgical techniques, and treatment outcomes. For instance, the UCI exemplifies how to successfully implement MDTs, integrating surgeons, oncologists, radiologists, and pathologists [26]. This model has significantly improved treatment coordination and patient outcomes, serving as a valuable example that other countries can adopt to enhance their cancer care systems.

Furthermore, patient education should be prioritized and expanded. The development of comprehensive patient education programs can empower individuals, improve their understanding of the disease and treatment options, and actively involve them in the decision‐making process. These programs should encompass important aspects of postoperative care, pain management, wound care, rehabilitation exercises, and community awareness campaigns to ensure well‐informed and engaged patients. Additionally, SSA can benefit from adopting guidelines such as those from the National Comprehensive Cancer Network (NCCN), European Society for Medical Oncology (ESMO), and National Institute for Health and Care Excellence (NICE). These guidelines provide comprehensive recommendations for the treatment of STS, covering surgical resection, adjuvant therapies, chemotherapy, radiotherapy, and management of metastatic disease [27, 28, 29]. Adapting these guidelines to local contexts and resources could enhance the quality of care and improve patient outcomes in the region.

Tailoring interventions based on insights gathered from patients, clinicians, and policymakers is crucial. Conducting surveys and interviews to gather feedback and perspectives can provide valuable information for designing interventions that address the specific needs and challenges of the region. Educational materials should be developed at appropriate literacy levels, and the involvement of caregivers and advocates should be emphasized to ensure effective patient comprehension and engagement. Moreover, exploring and developing affordable treatment options is necessary to mitigate the financial burden on STS patients. Implementing strategies to reduce treatment costs and facilitating access to necessary care should be prioritized. Furthermore, establishing efficient transportation networks can minimize travel costs and time burdens for patients seeking surgical management, ultimately improving accessibility to healthcare facilities.

**Figure 2:**
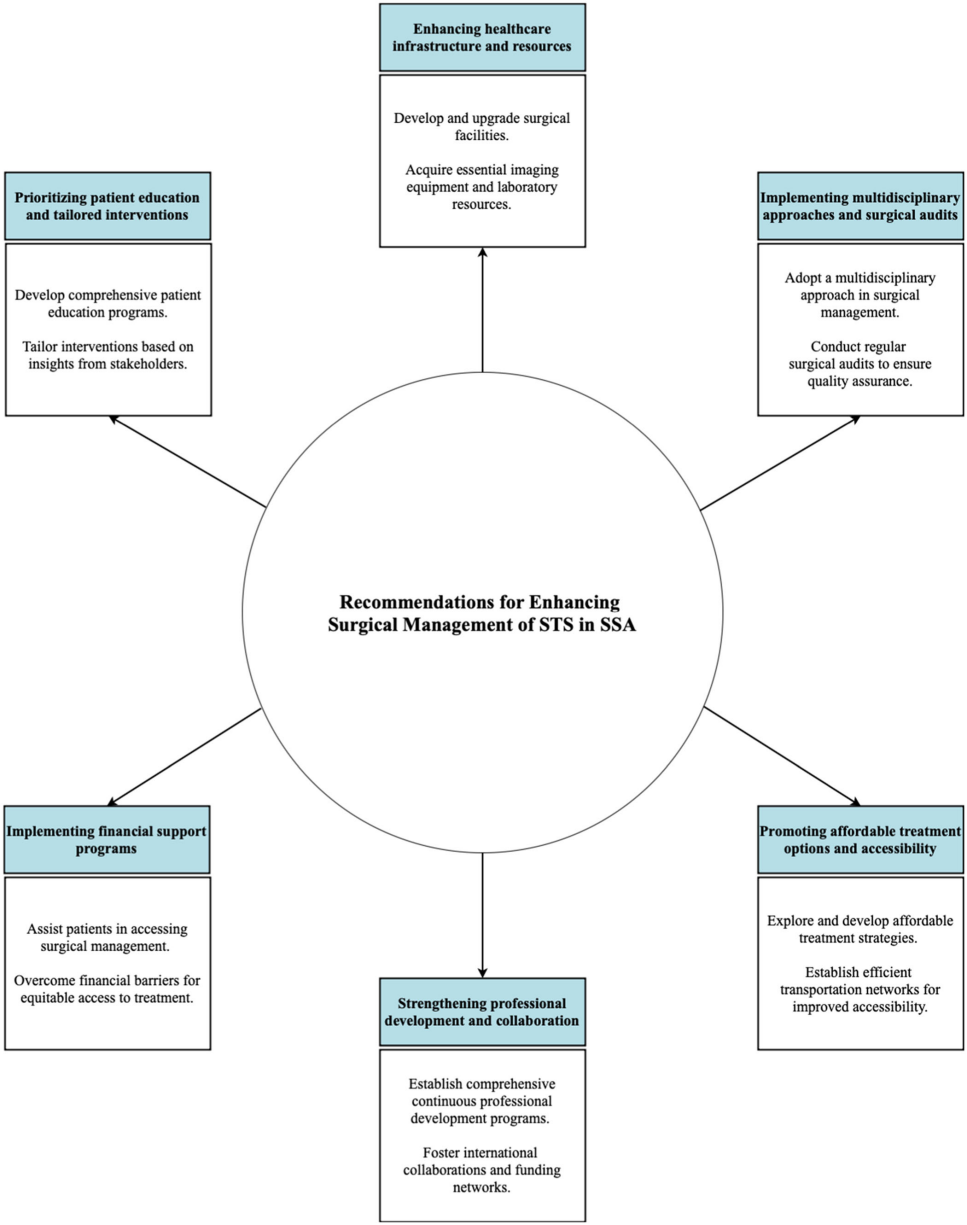
Recommendations for enhancing surgical management of STS in SSA.

Finally, implementing financial support programs to assist patients in accessing surgical management is of utmost importance. These programs can alleviate financial barriers and ensure equitable access to treatment for all STS patients, irrespective of their socioeconomic background. Future recommendations and prospects are summarized in Figure 2.

### 4.1 Limitations

This paper, while striving to offer a comprehensive overview of STS management in SSA, is inevitably constrained by several limitations. One limitation is the complexity of the regional context within SSA, which includes a diverse range of healthcare systems, resources, and cultural practices. These variations impact access to medical services, attitudes toward treatment, and overall management of STS. Therefore, it is crucial to acknowledge that strategies and recommendations must be adapted to these diverse contexts.

Another limitation is the reliance on available literature and data related to STS management. The quality, completeness, and reliability of these sources vary, which may affect the accuracy and robustness of our analysis. Additionally, retrospective data and small sample sizes in several studies may impact the generalizability of the findings. Furthermore, the authors' backgrounds, experiences, and biases may subtly influence the perspectives and interpretations presented, despite efforts to maintain objectivity.

A specific limitation noted is that the studies cited do not appear to include control groups, making it difficult to attribute improvements in outcomes solely to surgical interventions. Moreover, the predominance of English language sources may exclude valuable information from researchers unable to publish in English‐language journals, potentially limiting the paper's inclusivity.

## 5 Conclusions

The surgical management of STS in SSA demonstrates both favorable outcomes and notable challenges. While surgical resection remains crucial for achieving desirable results, the region encounters limitations concerning complete resection, high rates of local recurrence, postoperative complications, and mortality. Additionally, areas such as multidisciplinary care, access to specialized centers, workforce adequacy, comprehensive training, and research advancements demand focused attention. To address these gaps, recommendations include the enhancement of professional development, investment in healthcare infrastructure, adoption of a multidisciplinary approach, and improvement of patient education. Implementation of these measures holds the potential to enhance the surgical management and overall care of STS in SSA, thereby improving regional patient outcomes.

## Author Contributions

**Favour Tope Adebusoye:** conceptualization; writing–original draft; writing–review & editing. **Sakshi Roy:** writing–original draft; writing–review & editing. **Hareesha Rishab Bharadwaj:** writing–original draft; writing–review & editing. **Syed Hasham Ali:** writing–original draft; writing–review & editing. **Wireko Andrew Awuah:** writing–original draft; writing–review & editing. **Tomas Ferreira:** writing–original draft; writing–review & editing. **Joecelyn Kirani Tan:** writing–original draft; writing–review & editing. **Nicholas Aderinto:** writing–original draft; writing–review & editing. **Saleha Azeem:** writing–original draft; writing–review & editing. **Paul Olutosin Salako:** writing–original draft; writing–review & editing. **Pearl Ohenewaa Tenkorang:** writing–original draft; writing–review & editing. **Harendra Kumar:** writing–original draft; writing–review & editing. **Toufik Abdul‐Rahman:** writing–original draft; writing–review & editing. All authors have read and approved the final version of the manuscript.

## Ethics Statement

The authors have nothing to report.

## Conflicts of Interest

The authors declare no conflicts of interest.

## Transparency Statement

The lead author Wireko Andrew Awuah affirms that this manuscript is an honest, accurate, and transparent account of the study being reported; that no important aspects of the study have been omitted; and that any discrepancies from the study as planned (and, if relevant, registered) have been explained.

## Data Availability

The authors confirm that the data supporting the findings of this narrative review are available within the article and its supplementary materials. No additional data or materials are available beyond what is presented in the review. Wireko Andrew Awuah, the corresponding author, had full access to all of the data in this study and takes complete responsibility for the integrity of the data and the accuracy of the data analysis.
